# Cystic fibrosis transmembrane conductance regulator (CFTR) modulators have differential effects on cystic fibrosis macrophage function

**DOI:** 10.1038/s41598-018-35151-7

**Published:** 2018-11-20

**Authors:** Shuzhong Zhang, Chandra L. Shrestha, Benjamin T. Kopp

**Affiliations:** 1 Center for Microbial Pathogenesis, The Research Institute at Nationwide Children’s Hospital, Columbus, OH USA; 2 Division of Pulmonary Medicine, Nationwide Children’s Hospital, Columbus, OH USA

## Abstract

Despite the addition of cystic fibrosis transmembrane conductance regulator (CFTR) modulators to the cystic fibrosis (CF) treatment regimen, patients with CF continue to suffer from chronic bacterial infections that lead to progressive respiratory morbidity. Host immunity, and macrophage dysfunction specifically, has an integral role in the inability of patients with CF to clear bacterial infections. We sought to characterize macrophage responses to CFTR modulator treatment as we hypothesized that there would be differential effects based on patient genotype. Human CF and non-CF peripheral blood monocyte-derived macrophages (MDMs) were analyzed for CFTR expression, apoptosis, polarization, phagocytosis, bacterial killing, and cytokine production via microscopy, flow cytometry, and ELISA-based assays. Compared to non-CF MDMs, CF MDMs display decreased CFTR expression, increased apoptosis, and decreased phagocytosis. CFTR expression increased and apoptosis decreased in response to ivacaftor or lumacaftor/ivacaftor therapy, and phagocytosis improved with ivacaftor alone. Ivacaftor restored CF macrophage polarization responses to non-CF levels and reduced *Pseudomonas aeruginosa* bacterial burden, but did not reduce other bacterial loads. Macrophage inflammatory cytokine production decreased in response to ivacaftor alone. In summary, ivacaftor and lumacaftor/ivacaftor have differential impacts on macrophage function with minimal changes observed in CF patients treated with lumacaftor/ivacaftor. Overall improvements in macrophage function in ivacaftor-treated CF patients result in modestly improved macrophage-mediated bacterial killing.

## Introduction

The emerging use of cystic fibrosis (CF) transmembrane conductance regulator (CFTR) modulators has provided CF patients with unprecedented access to potentially life-extending therapies^1^. In 2015 the United States Food and Drug Administration approved lumacaftor/ivacaftor for patients with the p.Phe508del (F508del) mutation who have minimal plasma membrane CFTR expression. While lumacaftor/ivacaftor diminishes pulmonary exacerbations and risk of hospitalization in CF patients^2^, when compared to ivacaftor alone (a CFTR modulator of gating mutations)^3^, lumacaftor/ivacaftor is significantly less effective in improving two key outcomes, lung function and body mass index (BMI). In addition, the long-term clinical significance of lumacaftor/ivacaftor and its influence on chronic inflammation and clearance of chronic bacterial infections in CF patients are not established, and those of ivacaftor are limited. Furthermore, despite the clinical benefits achieved by CFTR modulators, CF patients continue to suffer from chronic bacterial infections and heightened inflammation leading to recurrent sinopulmonary morbidity and ultimately mortality. In particular, *Pseudomonas aeruginosa* infections persist in patients with CF on Ivacaftor^4^ despite other perceived clinical benefits.

Previous research in CF infection control focused on new antimicrobials and anti-inflammatory agents and often did not address host deficits in immunological responses. In the past decade, studies by our group and others^5–16^ indicate that host immune cells, and particularly macrophages, have an integral role in clearance of chronic bacterial infections in CF patients. We have shown that CF macrophages can serve as a replicative niche for bacteria to avoid host defenses^5^, have deficits in bacterial killing^5,9,12^, and produce excess cytokines in response to infection^6^.

To understand if treatment with CFTR modulators could alter CF macrophage responses to infection, we sought to characterize macrophage responses to clinical treatment with CFTR modulators. Based on differences in existing clinical trial outcomes, we hypothesized that there would be differential effects of CFTR modulators upon macrophage function dependent on patient genotype.

## Results

### Patient Demographics

The demographics of the patients who contributed to this study are listed in Table 1 and their genotypes in Supplemental Table 1. Non-CF patients were slightly older than the CF patients, but the 2 groups were overall similar in terms of age, gender, and ethnicity. Patients with CF had predominantly class I/II CFTR mutations (severe disease) excepting those on ivacaftor (class III/IV/V). Patients with CF had a mean BMI near 21 as an indicator of nutritional status, and an average percent predicted forced expiratory volume in 1 second (FEV_1_) of 68% as a marker of lung disease.

**Table 1:** Patient demographics.

	CF (n = 37 unique pts)	Non-CF (n = 42)	P value
Age (years)	27.14 ± 14.66	31.88 ± 11.05	0.11
Male	43.4%	47.6%	0.43
Caucasian	94.6%	85.7%	0.20
CFTR genotype			
Class I/II (n = 27)	73.0%		
Class III/IV/V (n = 10)	27.0%		
BMI (mean)	20.93 ± 4.73		
*FEV_1_ (%predicted)	64.25 ± 28.78		

*%predicted FEV_1_ < 80% are suggestive of airway obstruction.

P values determined by unpaired t-test.

### Macrophages from CF patients under CFTR modulation have altered CFTR expression and cell stability

**Figure 1:**
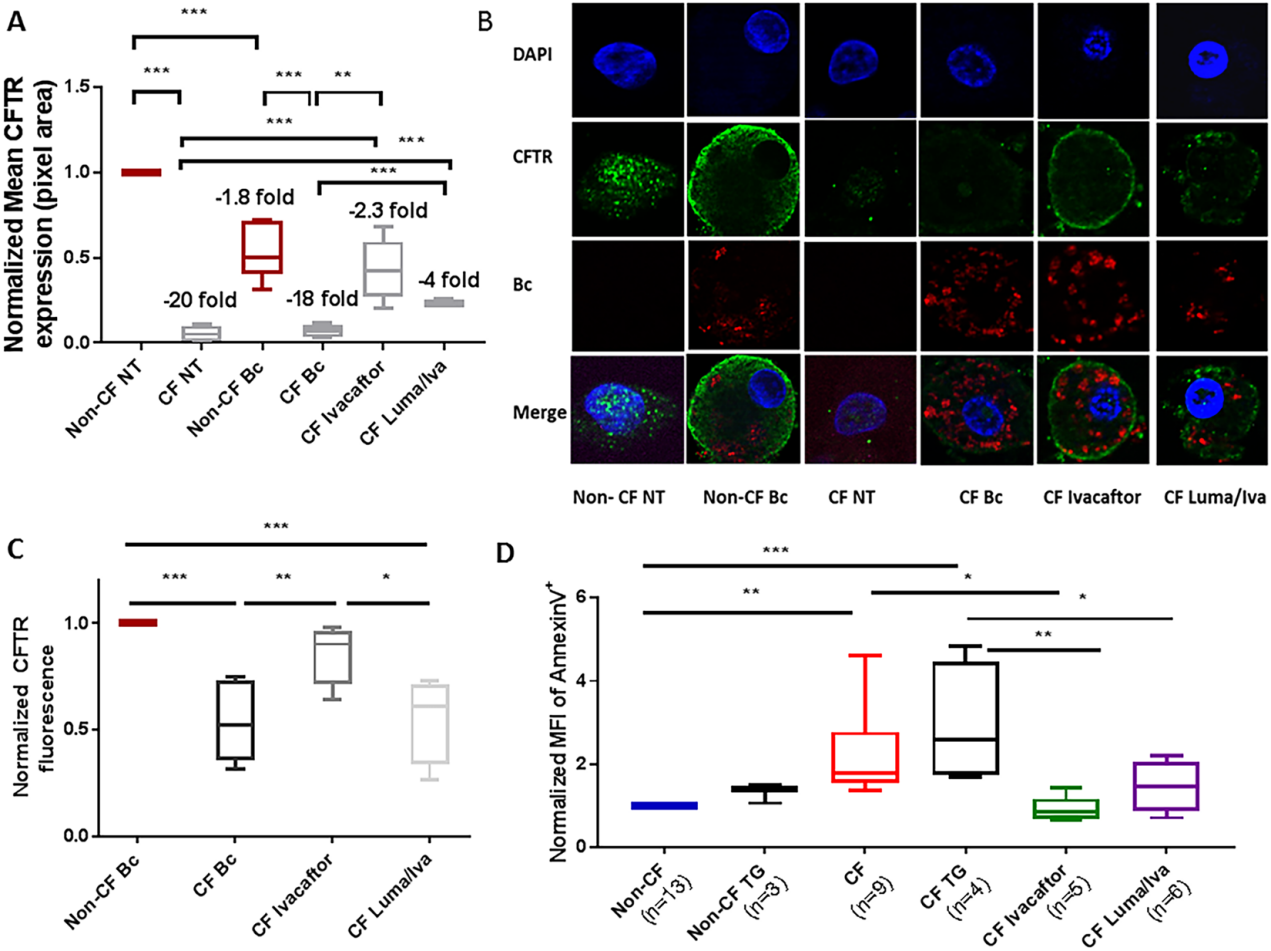
Macrophages from CF patients under CFTR modulation have altered CFTR expression and cell stability: (**A**) Mean CFTR expression was determined using ImageJ software of 5 independent fields of 10 MDMs for at least 4 patients per condition from fluorescent microscopy images, MOI 2. Fold change expression are presented in comparison to uninfected non-CF MDMs (non-CF NT). MDMs were grouped according to CF (F508del/F508del or F508del/G551D) and non-CF patients with or without infection with *B*. *cenocepacia* (Bc) and on clinical treatment with CFTR modulators (lumacaftor/ivacaftor or ivacaftor). CFTR modulator groups were infected with Bc. “NT” denotes no infection. “*” denotes a p value < 0.05, “**” denotes a p value < 0.01, and “***” denotes a p value < 0.001, unpaired t-test. (**B**) Representative image of fluorescent microscopy of MDMs from (**A**). CFTR is expressed in green, bacteria in red, and the macrophage nucleus is stained blue with DAPI. n = 4–6. CFTR modulator groups were infected with Bc. “NT” denotes no infection. (**C**) CFTR mean fluorescence detected via flow cytometry in human CF MDMs on or off CFTR modulators and normalized to non-CF MDMs, all groups with infection with *B*. *cenocepacia* (Bc). “**” denotes a p value < 0.01, and “***” denotes a p value < 0.001, one-way ANOVA with post-hoc Tukey. (**D**) Apoptosis was measured via an Annexin V detection assay via flow cytometry, and normalized to non-CF MDMs. Groups included non-CF MDMs (n = 13) and CF MDMs (n = 9) at baseline, in response to the apoptosis inducer thapsagargin (TG) (n = 3–4), and CF MDMs from patients on CFTR modulators (n = 5–6). “**” denotes a p value < 0.01, and “***” denotes a p value < 0.001, one-way ANOVA with post-hoc Tukey.

Multiple groups have demonstrated that human and pig CF macrophages have decreased CFTR expression^12,17–20^. To determine if CFTR modulators can increase macrophage CFTR expression we performed fluorescent microscopy using human CF and non-CF monocyte-derived macrophages (MDMs). CFTR expression was greater in non-CF MDMs at baseline compared to CF (Fig. 1A,B). During infection with *B*. *cenocepacia*, CFTR was noted to re-organize to the peripheral membrane in non-CF but not CF MDMs, similar to our prior study^12^ (Fig. 1B). CFTR expression also remained significantly greater in non-CF MDMs during infection compared to CF (Fig. 1A,B). CFTR expression significantly increased during infection in CF patients taking either ivacaftor or lumacaftor/ivacaftor compared to untreated CF patients, but did not reach non-CF levels (Fig. 1A,B). CFTR expression in non-CF MDMs was unchanged by *in-vitro* treatment with CFTR modulators (not shown). Recently, the specificity of certain antibodies to detect CFTR in epithelial cells was called into question^21^. To confirm the specificity of the anti-CFTR [CF3] (ab2784) antibody used for immunofluorescence, we tested the CF3 antibody in a CRISPR-Cas9 system separately developed to knockout CFTR in non-CF MDMs. CFTR was readily detected by Western blot in vehicle-transfected non-CF MDMs, while decreased CFTR expression was shown in CRISPR-Cas9 CFTR knockout MDMs and confirmed by RT-PCR (Fig. S1A–D). We then used a flow cytometry-based assay^13^ to quantify CFTR in human CF and non-CF MDMs infected with *B*. *cenocepacia* via the CF3 antibody (Fig. 1C), which confirmed the findings from Fig. 1A,B. CFTR expression results were similar when comparing the CF3 antibody to anti-CFTR-596 and anti-CFTR-570 obtained from the CFTR Antibody Distribution Program (https://www.cff.org/Research/Researcher-Resources/Tools-and-Resources/CFTR-Antibodies-Distribution-Program/). It was noted that for some patients CFTR-570 demonstrates high background fluorescence. A representative flow cytometry gating output diagram comparing all 3 antibodies for a CF patient not on CFTR modulators compared to a CF patient on Ivacaftor and a non-CF patient is displayed (Fig. S2A) along with summed data for non-CF and CF MDMs (Fig. S2B).

In order to determine if the observed changes in CFTR expression were associated with changes in macrophage stability, we used flow cytometry to measure cellular apoptosis. Untreated CF MDMs demonstrated a 2-fold or greater rate of apoptosis in comparison to non-CF MDMs (Figs 1D, S1E). There was a similar pattern of increased apoptosis in untreated MDMs compared to non-CF MDMs in response to the apoptosis inducer thapsagargin (Fig. 1D). CF patients on ivacaftor had similar levels of macrophage apoptosis to non-CF patients, while patients on lumacaftor/ivacaftor had a non-significant decrease in apoptosis in comparison to untreated CF MDMs (Fig. 1D). Taken together, these findings show that CFTR modulators can improve macrophage CFTR expression, which is associated with decreased cellular apoptosis.

### Macrophages from CF patients on Ivacaftor have altered polarization

**Figure 2:**
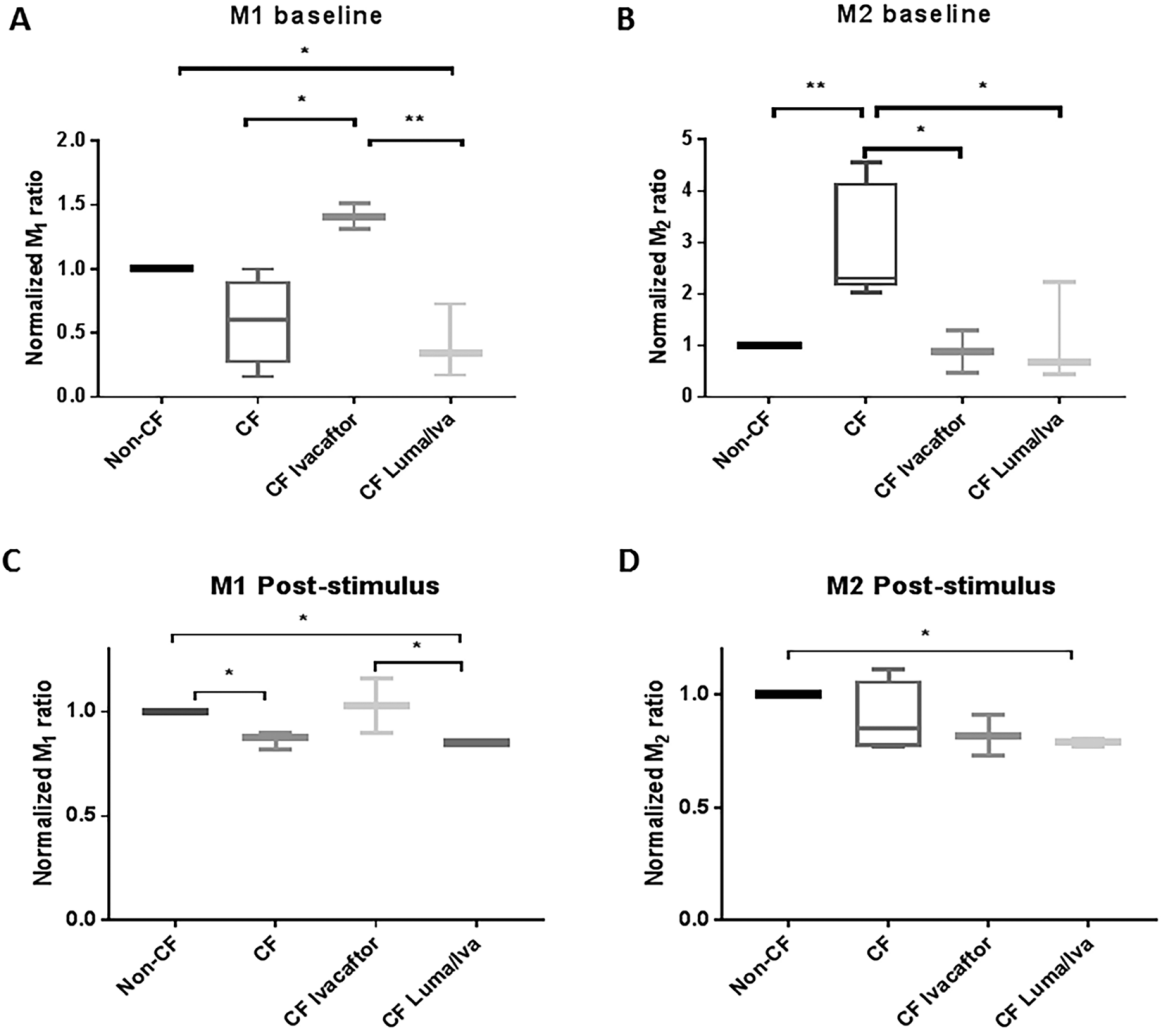
Macrophages from CF patients on Ivacaftor have altered polarization. (**A**) Baseline M1 and (**B**) M2 polarization of freshly isolated non-CF and CF monocytes. CF monocytes were grouped according to the presence or absence of clinical CFTR modulator treatment. “*” denotes a p value < 0.05 and “**” denotes a p value < 0.01, one-way ANOVA with post-hoc Tukey. (**C**) M1 and D) M2 polarization of non-CF and CF MDMs after 48 h exposure to polarizing stimulus. Data were normalized to non-CF MDMs. “*” denotes a p value < 0.05, one-way ANOVA with post-hoc Tukey. The flow cytometry gating strategy for Fig. 2 is found online (Supplementary Fig. S1), M1: CD68/CD80; M2: CD163/CD206.

Macrophages exhibit functional plasticity and can exist in a pro-inflammatory (M1) or an alternatively activated anti-inflammatory/pro-fibrotic (M2) state^22^. The ability of CF macrophages to respond to M1 and M2 polarizing stimuli is controversial and differs among studies^14,23–25^. We determined baseline polarization in freshly isolated monocytes as well as the ability of differentiated MDMs to respond to polarizing stimuli. At baseline, CF monocytes demonstrated a high M2 and low M1 phenotype in comparison to non-CF monocytes (Fig. 2A,B). CF patients taking ivacaftor had similar levels of M1 and M2 polarized monocytes to non-CF (Fig. 2A,B). CF patients taking lumacaftor/ivacaftor had persistently low levels of M1 polarized monocytes and comparable levels of M2 (Fig. 2A,B). There was a similar profile for CF MDMs in response to an M1 stimulus, with low levels of M1 CF MDMs compared to non-CF, restoration with ivacaftor, and failure to change with ivacaftor/lumacaftor (Fig. 2C). In contrast, there was no difference in levels of M2 MDMs except in CF patients on lumacaftor/ivacaftor who had significantly less M2 MDM polarized cells in comparison to non-CF MDMs. A representative figure of the flow cytometry gating strategies for Fig. 2 is found online in Supplementary Fig. S3. Combined, these results suggest that the activation state of CF monocytes and macrophages is altered at baseline and in response to stimuli, but is responsive to CFTR modulator therapy.

### Macrophages from patients on Ivacaftor have improved phagocytosis and killing of *Pseudomonas aeruginosa*

**Figure 3:**
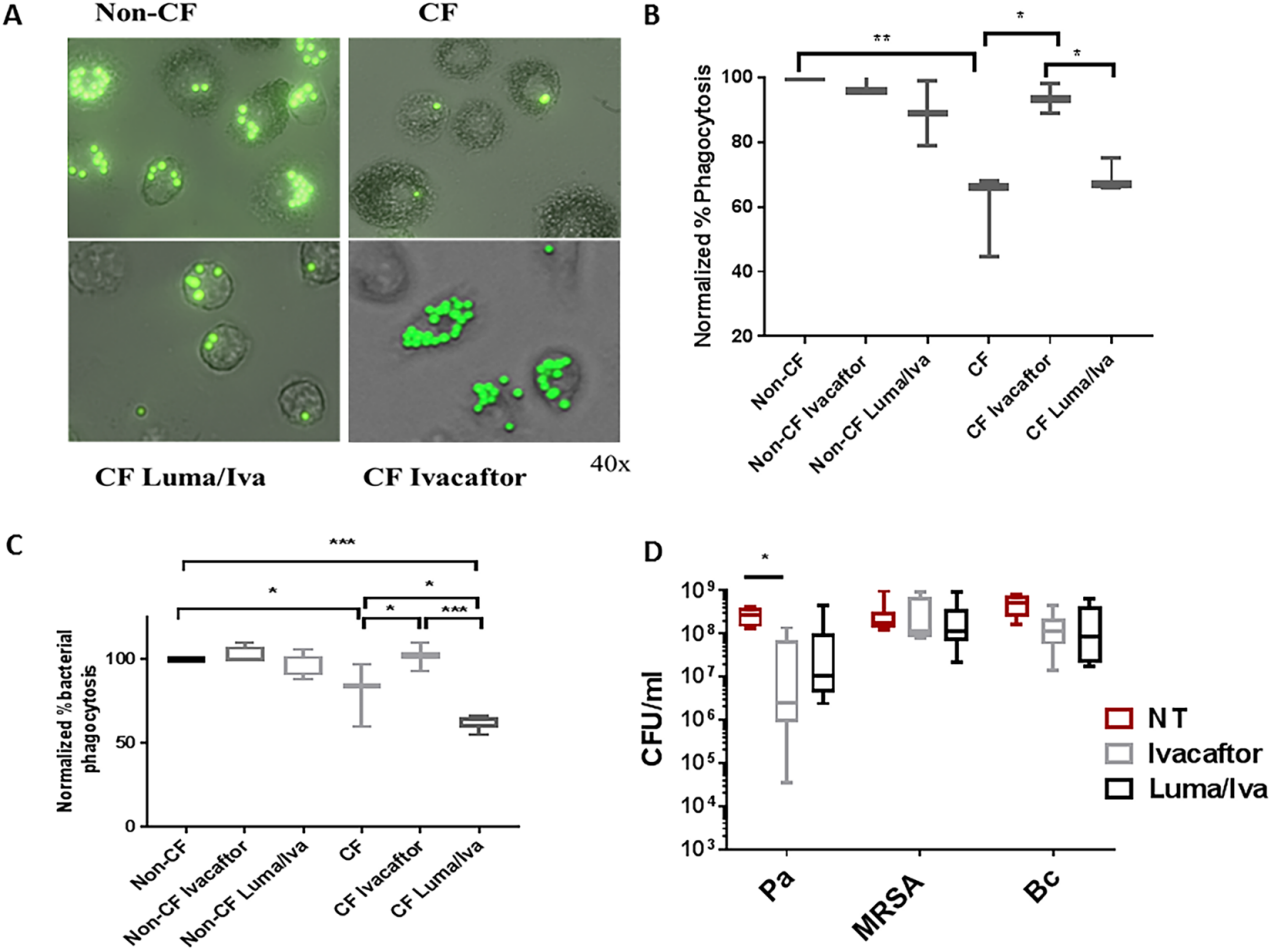
Macrophages from patients on Ivacaftor have improved phagocytosis and killing of Pseudomonas aeruginosa. (**A**) Representative microscopy image of non-CF and CF MDM phagocytosis of FITC-labeled beads, 40X magnification, MOI 50. CF MDMs were grouped according to the presence or absence of clinical CFTR modulator treatment. (**B**) Summed % phagocytosis normalized to non-CF MDMs for (**A**) “*” denotes a p value < 0.05 and “**” denotes a p value < 0.01, one-way ANOVA with post-hoc Tukey. (**C**) Summed %phagocytosis of RFP-expressing *B*. *cenocepacia* normalized to non-CF MDMs, n = 3–9/group, MOI 50. “*” denotes a p value < 0.05, “**” denotes a p value < 0.01, and “***” denotes a p value < 0.001, one-way ANOVA with post-hoc Tukey. (**D**) Colony-forming unit (CFU) assay for CF MDMs Infected with *P*. *aeruginosa* (Pa), MRSA, and *B*. *cenocepacia* (Bc). CF MDMs were grouped according to the presence or absence of clinical CFTR modulator treatment, n = 4–8/group, one-way ANOVA with post-hoc Tukey.

CF macrophages have altered phagocytosis and decreased intracellular killing of bacteria such as *B*. *cenocepacia* and *P*. *aeruginosa*^5,12,26,27^. In order to determine the impact of CFTR modulators on phagocytosis and bacterial killing, we first measured phagocytosis using fluorescent beads and then using RFP-expressing *B*. *cenocepacia*. CF MDMs had a 40% or greater reduction in phagocytosis of beads compared to non-CF MDMs (Fig. 3A,B). CF patients taking ivacaftor, but not lumacaftor/ivacaftor, demonstrated restoration of phagocytosis levels compared to non-CF MDMs (Fig. 3A,B). *In-vitro* treatment with CFTR modulators did not significantly change phagocytosis in non-CF MDMs, but we noted a trend towards decreased phagocytosis with lumacaftor/ivacaftor treatment (Fig. 3B). CF MDMs also demonstrated decreased phagocytosis of *B*. *cenocepacia* compared to non-CF MDMs (Fig. 3C). CF patients on ivacaftor had significant increases in *B*. *cenocepacia* phagocytosis compared to CF patients not on CFTR modulators (Fig. 3C). Interestingly, CF patients on lumacaftor/ivacaftor had decreased *B*. *cenocepacia* phagocytosis compared to CF patients not on CFTR modulators and non-CF patients (Fig. 3C).

Due to the changes in phagocytosis observed, we then measured macrophage-mediated bacterial killing of three virulent CF pathogens (*P*. *aeruginosa*, MRSA, and *B*. *cenocepacia*) in response to CFTR modulators. Patients with CF had no differences in MRSA or *B*. *cenocepacia* bacterial load whether on or off CFTR modulators, although on average *B*. *cenocepacia* load was reduced by one log for both modulators (Fig. 3D). There was a significant 89% decrease in *P*. *aeruginosa* bacterial load in ivacaftor-treated CF patients and a non-significant reduction in lumacaftor/ivacaftor-treated patients (Fig. 3D). Combined, these findings suggest that CFTR modulators can restore phagocytosis, but improved phagocytosis results in differentially enhanced macrophage-mediated bacterial killing.

### Macrophages from CF patients on CFTR modulators have differential cytokine production

**Figure 4:**
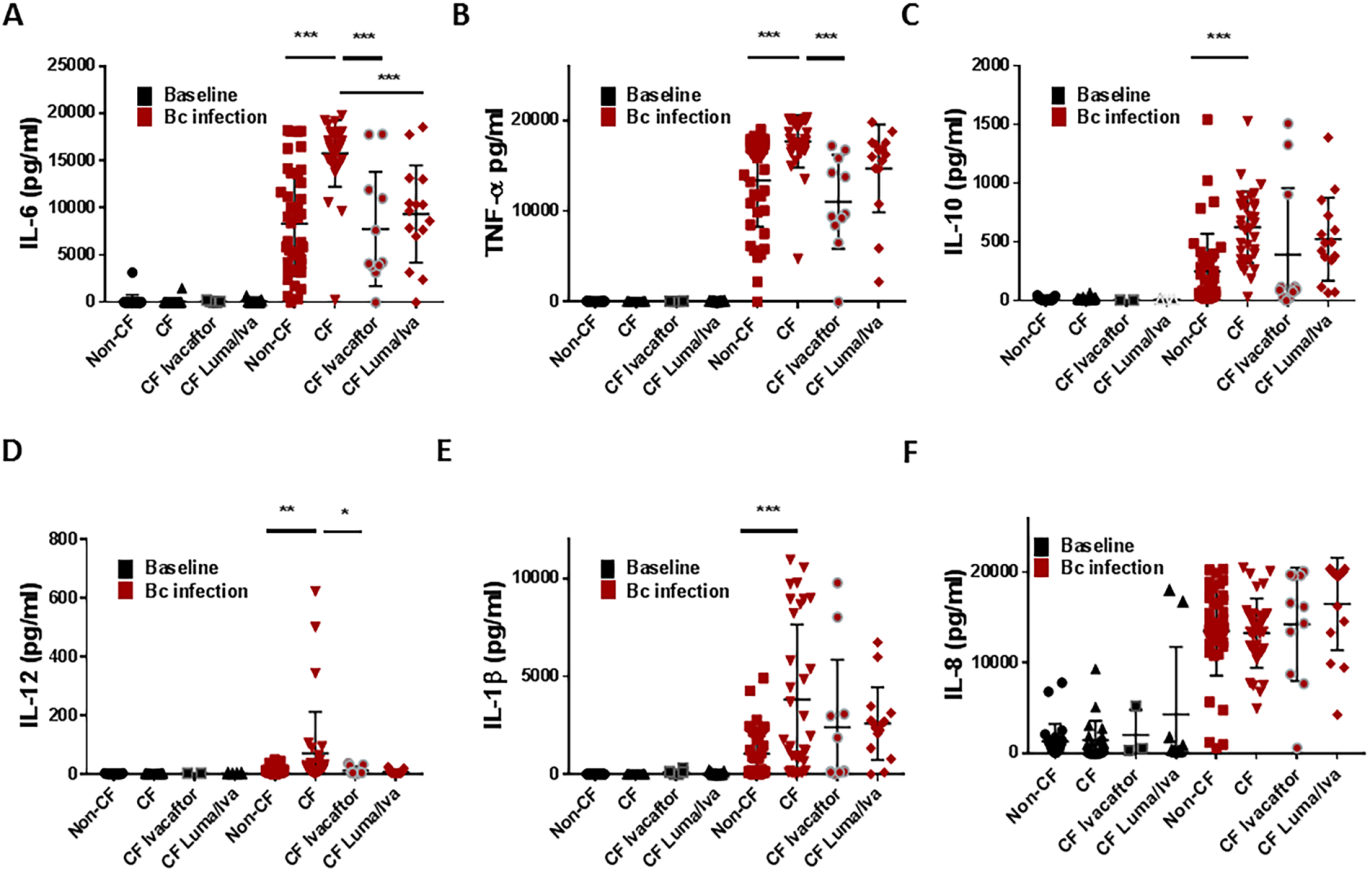
Macrophages from CF patients on CFTR modulators have differential cytokine production. Multiplex cytokine assay analysis of CF (n = 33) and non-CF (n = 41) MDM supernatants after 24 h with or without infection with *B*. *cenocepacia* (Bc, MOI 10) and/or treatment with ivacaftor or lumacaftor/ivacaftor (luma/iva) for (**A**) IL-6, (**B**) TNF-α, (**C**) IL-10, (**D**) IL-12, (**E**) IL-1β, and (**F**) IL-8. “*” denotes a p value < 0.05, “**” denotes a p value < 0.01, and “***” denotes a p value < 0.001, one-way ANOVA with post-hoc Tukey.

CF macrophages are characterized by a hyper-inflammatory state^14,15^, especially during infection with pathogens such as *B*. *cenocepacia*^6^. We measured a panel of cytokines in human CF and non-CF MDM supernatants from patients on or off CFTR modulators at baseline and in response to *B*. *cenocepacia* infection (Fig. 4). There were no major differences in cytokine levels at baseline between non-CF, CF, and treated CF MDMs (Fig. 4A–F). There were significant increases in IL-6, TNF-α, IL-10, IL-12, and IL-1β concentrations in CF MDMs after *B*. *cenocepacia* infection compared to non-CF (Fig. 4A–E). Treatment with ivacaftor reduced IL-6, TNF-α, and IL-12 production in CF MDMs to levels comparable to non-CF (Fig. 4A,B,D). In contrast, treatment with lumacaftor reduced IL-6 production only in CF MDMs (Fig. 4A), although a trend in reduction was noted for IL-12. There was no difference in IL-8 production between any of the groups (Fig. 4F). Overall, we noted some variability in individual cytokine responses for both CFTR modulators.

**Figure 5:**
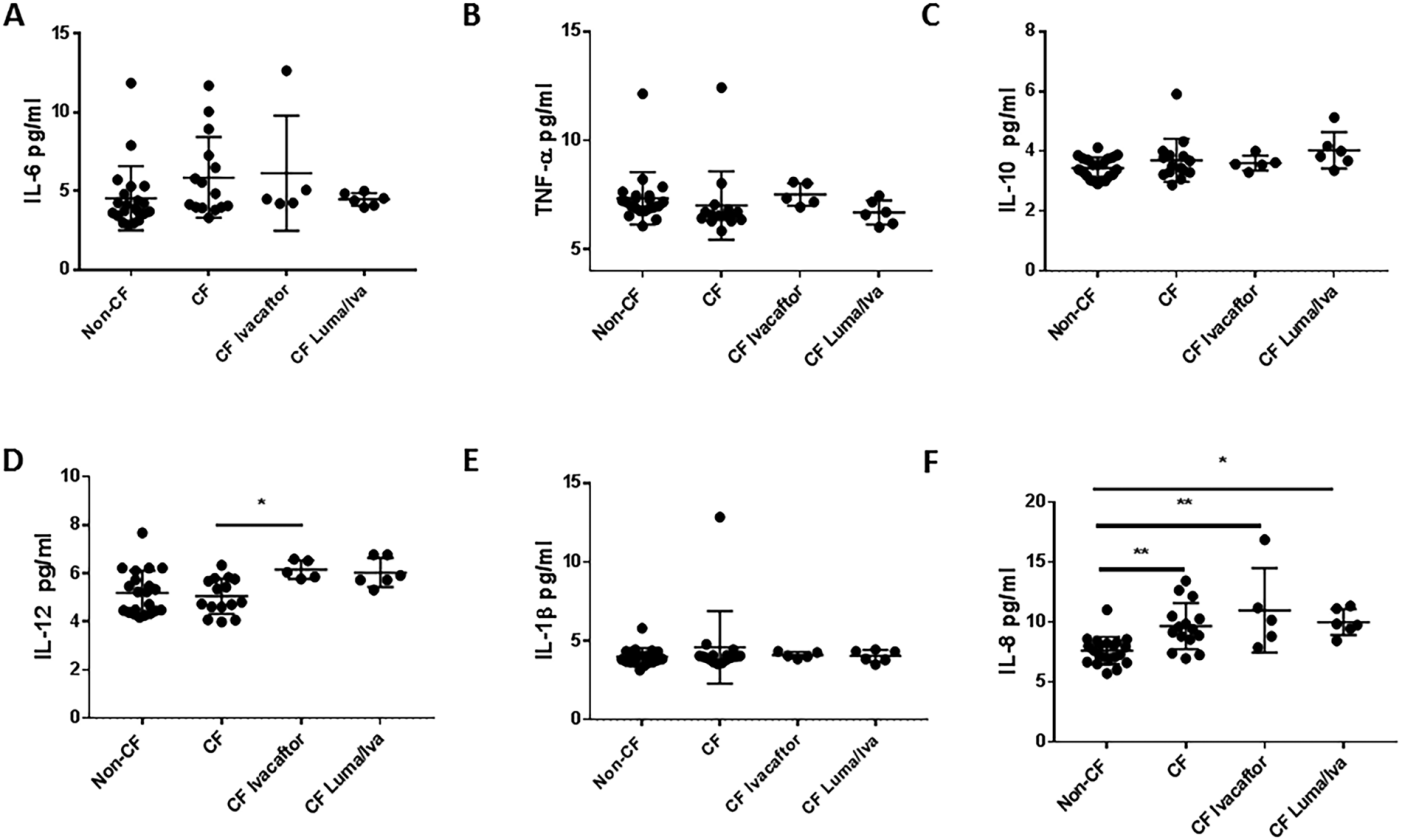
CFTR modulators do not change serum cytokine production. Multiplex cytokine assay analysis of CF (n = 15) and non-CF serum (n = 21) in the presence or absence of ivacaftor or lumacaftor/ivacaftor (luma/iva) for (**A**) IL-6, (**B**) TNF-α, (**C**) IL-10, (**D**) IL-12, (**E**) IL-1β, and (**F**) IL-8. “*” denotes a p value < 0.05, “**” denotes a p value < 0.01, and “***” denotes a p value < 0.001, one-way ANOVA with post-hoc Tukey.

To examine if differences existed in systemic cytokine production, we measured the same cytokines in serum isolated at the time of fresh monocyte collection (Fig. 5). There were no significant differences in any serum cytokine levels between groups except for higher levels of IL-8 in CF at baseline and post-CFTR modulators compared to non-CF (Fig. 5F), and higher IL-12 production in CF patients on ivacaftor compared to no treatment (Fig. 5D). Additionally, when comparing individual paired serum cytokine changes between CF patients pre- and post-CFTR modulators, IL-8 did not significantly change for either CFTR modulator group post-treatment (Fig. S4). There were significant increases in IL-12 for both CFTR modulator groups post-treatment, as well an increase in TNF-α post-ivacaftor (Fig. S4). Together, macrophage and serum cytokine responses suggest inflammatory responses to bacteria are altered with CFTR modulator treatment, while baseline cytokine production either at the systemic or cellular level is a poor indicator of response to treatment.

## Discussion

Cystic fibrosis remains an under-recognized immunodeficiency due to dysfunctional CFTR that leads to the production of dehydrated mucus, impaired immunity, and subsequent acute and chronic bacterial infections that cause progressive structural and functional changes in the respiratory tract. Classically, chronic infection treatments in CF have focused on exaggerated neutrophil recruitment and the mucus environment that facilitates bacterial growth. Over the past decade, our knowledge base has expanded to include the role underlying deficits in macrophage and overall innate immune responses of patients with CF have in disease severity and progression^5,15,28–34^. However, no definitive cure for CF exists, including standard ways to improve immune-mediated bacterial clearance. Although advances in CF knowledge and care (i.e., CFTR modulators) have improved clinical outcomes^3,35^, patients with CF remain burdened by chronic, multi-drug-resistant bacterial infections. To this end, we determined functional responses of CF macrophages from patients undergoing CFTR modulation and identified deficits that persist despite CFTR modulator treatment. Incomplete resolution of deficits in CF macrophage function may in part explain why bacteria persist in CF patients during treatment with CFTR modulators.

Multiple independent groups have defintively established that CFTR is expressed in human macrophages^12,17–19^. The function of CFTR in macrophages remains less well-established. We demonstrated that CFTR is present in CF and non-CF macrophages and increases in expression during treatment with CFTR modulators, including a re-organization of CFTR to the peripheral membrane during infection. Increases in CFTR expression were correlated with decreased cellular apoptosis, with macrophages from ivacaftor-treated CF patients demonstrating more robust CFTR expression and decreased apoptosis compared to lumacaftor-ivacaftor treated patients. Differences in CFTR expression and apoptosis between CFTR modulator treatments suggests that CFTR has a role in macrophage stability, and that this response may depend on the amount of functional CFTR. We observe that as CFTR expression increases, cellular apoptosis decreases. CFTR ion efflux was not able to be tested in this study to determine if CFTR expression is associated with functional changes, but will be needed in the future to confirm our findings.

In conjunction with our findings that cellular apoptosis was improved, we discovered changes in monocyte and macrophage polarization in response to CFTR modulation. Previously it has been reported that human CF MDMs fail to respond to M2 polarizing stimuli^23^, macrophage activation phenotypes may be altered by medications such as azithromycin^24^, and that differences exist between murine and human macrophage responses^7^. We saw a similar pattern in our MDM responses to an M2 stimulus as most CF MDMs had decreased M2 activation, although there was a range of patient responses with some CF patients displaying normal M2 activation. The M2 activation responses were not improved by CFTR modulators. In contrast, in freshly isolated monocytes we saw a greatly increased percentage of M2 polarized cells, which were responsive to CFTR modulators. These results together suggest that CF macrophages may lose plasticity over time, with some cells persisting in an M2 activation state, with others losing the ability to differentiate. Concurrently, we also observed that both freshly isolated monocytes and MDMs had decreased M1 polarization, but were responsive to ivacaftor therapy. We note that our MDMs were from teenage and adult patients, and we use accutase detachment of macrophages prior to flow cytometry as we have found this to cause the least apoptosis compared to other agents containing trypsin or EDTA. Our results combined with the existing literature demonstrate that most macrophage activation phenotypes respond to CFTR modulation, but may be dependent on other factors such as length of culture, polarizing stimuli, age, medications, and macrophage environment.

Although macrophage activation states may vary between studies, CF monocyte and macrophage phagocytosis is defective across studies^14,16,26,27,36^. In confirmation of prior studies, we observed that that phagocytosis of both fluorescently labeled beads and bacteria was decreased in CF macrophages compared to non-CF. However, only macrophages from patients with ivacaftor-responsive mutations demonstrated improvements in phagocytosis of beads or bacteria, while lumacaftor/ivacaftor treatment did not improve phagocytosis in F508del homozygous CF patients. In fact, lumacaftor/ivacaftor worsened bacterial phagocytosis in CF patients. These findings may be explained by a recent study^27^ where it was shown that ivacaftor reduced the ability of lumacaftor to stimulate phagocytosis and killing of *P*. *aeruginosa*, which suggests that there may be negative effects of the drug combination on CF macrophage phagocytosis. Additionally, improvements in phagocytosis only decreased the bacterial load of *P*. *aeruginosa*, and not MRSA or *B*. *cenocepacia*. This suggests that either further increases in CFTR expression or activation are necessary for complete bacterial killing, macrophage responses unaffected by CFTR modulation may be involved, other immune cells are needed to enhance killing of bacteria, or some combination of the above.

Finally, we noted differential responses in macrophage pro- and anti-inflammatory cytokine production during infection with *Burkholderia cenocepacia*. Consistent with the majority of our other findings, MDMs from patients on ivacaftor displayed normalized cytokine production similar to non-CF MDMs. This may be reflective of a direct response to CFTR modulation or reduced bacterial burden. Interestingly, we did not discern differences in macrophage IL-8 production among groups, despite the known increase in IL-8 in CF airways. Our findings are likely explained by the absence of other IL-8 producing cells such as endothelial and epithelial cells, as well as the absence of IL-8’s main target, the neutrophil. Additionally, we observed varied cytokine responses on an individual basis within each CFTR modulator group. Variable responses suggest that not all individuals receive the full benefit of CFTR modulation upon macrophage function, whether due to non-compliance, co-morbidities, genetic modifiers, or other complicating factors.

Our study was limited by the use of blood monocytes and blood MDMs, which may have differential phenotypes compared to alveolar macrophages. However, recruited peripheral blood monocytes have an established role in lung disease^37^, and monocyte-derived AMs (MoAMs) were recently recognized to contribute to lung disease^38^. We also cannot account for other immune cells or circulating factors that may influence macrophage function in patients independent of a direct effect of CFTR modulation itself.

In summary, macrophages from CF patients on ivacaftor and lumacaftor/ivacaftor have differential responses with minimal changes observed in CF patients treated with lumacaftor/ivacaftor. Overall improvements in macrophage function seen in ivacaftor-treated CF patients modestly improve macrophage-mediated bacterial killing. Findings from this study should be the target of future work aimed at improving the killing of bacteria in patients with CF.

## Methods

### Human subjects

Human subjects were recruited as approved by the Institutional Review Board of Nationwide Children’s Hospital (IRB16-01020). All experiments were performed in accordance with relevant guidelines and regulations. Study subjects provided written informed consent for procedures if of legal age, and children provided written informed assent and a parent or guardian of any child participant provided informed consent on their behalf. Clinical information was recorded from clinic visits into a REDCap database.

### Macrophage isolation

Heparinized blood samples were obtained from patients with CF and age and gender-matched non-CF healthy controls. Subjects were excluded if using chronic immunosuppressants or if they had a history of transplantation. Peripheral monocytes were isolated from whole blood using Lymphocyte Separation Medium (Corning, 25-072-CV). Isolated monocytes were re-suspended in RPMI (Gibco, 22400-089) plus 10% human AB serum (Corning, 35-060-Cl) and differentiated for 5 days at 37 °C into MDMs^6,39^. MDMs were confirmed by microscopy and flow cytometry. MDMs were then placed in a monolayer culture, and infected at bacterial multiplicity of infection (MOI) ranging from 2–50.

### Bacterial strains and colony forming unit assay

Macrophages were infected with RFP-expressing *B*. *cenocepacia* strain k56-2, a methicillin-resistant *Staphylococcus aureus* (MRSA) isolate obtained from a CF patient’s sputum, or a MDR *Pseudomonas aeruginosa* isolate obtained from a CF patient’s sputum. The *B*. *cenocepacia* strain is representative of an epidemic clinical strain from the ET12 lineage^40^. Bacteria were reproducibly grown in LB media over 24 h. Colony forming unit (CFU) analysis was performed as previously described^12^. Recovered bacteria were quantified by plating serial dilutions on LB agar plates and analyzed for CFUs.

### Microscopy

One million macrophages were cultured on 12 mm glass cover slips in 24-well tissue culture plates and infected synchronously with *B*. *cenocepacia* at an MOI of 2 due to clumping at higher MOIs. Macrophage nuclei were stained blue with DAPI. CFTR was detected with CFTR antibody (2784, Abcam, CF3) followed by fluorescent secondary antibodies (Molecular Probes, A11008). Microscopy was performed using an Axiovert 200 M inverted epifluorescence microscope equipped with the Apotome attachment for improved fluorescence resolution and an Axiocam MRM CCD camera (Carl Zeiss Inc., Thornwood, NY). Five independent fields with at least 10 macrophages were scored for each condition. All experiments were performed in triplicate.

### CRISPR-Cas9 CFTR knockout for antibody validation

One to two million MDMs were placed in 12-well tissue culture plates in 1 ml of antibiotic-free RPMI medium with 10% AB serum per well. MDMs were rested for 4 hours prior to transfection. MDMs were then transfected with a CFTR CRISPR/Cas9 knockout plasmid (sc-400653, Santa Cruz) and a CFTR homology-directed repair (HDR) plasmid (sc-400653-HDR, Santa Cruz). A plasmid solution (A) was prepared by diluting plasmids (1.5 μg) to a final volume of 100ul in transfection medium (sc-108062, Santa Cruz). A transfection solution (B) was prepared by diluting 6 μl of UltraCruz® transfection reagent (sc-395739) with 94 μl transfection medium. Solution A was added dropwise directly to solution B and vortexed immediately. This mixture (C) was incubated for 30 minutes at room temperature. Then, 800 μl transfection media was added to the combined mixture (C) and the solution gently pipetted. Solution C was washed twice with RPMI and then added dropwise to the MDMs in the 12-well tissue culture plate. The plate was then incubated for 6 hours. After 6 hours, transfected MDMs were given fresh media and incubated for another 20 hours. Finally, the supernatant was aspirated and replaced with fresh media containing puromycin at 2 μg/ml for additional 24 hours before harvesting transfected MDMs for protein or RNA extraction. RNA was extracted from MDMs using the Total RNA Purification Kit (Norgen Biotek, 17200). Semi-quantitative RT-PCR was then performed by synthesizing cDNA by reverse transcriptase (Santa Cruz, sc43500) and then using nested PCR via two pairs of CFTR primer (CFTR (h)-PR, Santa Cruz, sc35054-PR) for determining CFTR transcription level. 18S rRNA (Forward: 5′-GGTGAAATTCTTGGACCGGC-3′; Reverse:5′-GACTTTGGTTTCCCGGAAGC-3′) was used as an internal control.

### Intracellular staining of CFTR by Flow-Cytometry

Macrophage (0.8e6/per condition) were fixed for 20 minutes with 1 ml of fixation buffer (Invitrogen, 00822249) at room temperature (RT) in a FACS tube, washed twice with cold PBS, and then 1 mL permeablization buffer (Invitrogen, 00833356) was added for 15 minutes at RT. Cells were then centrifuged at 1400 rpm × 5 minutes at 4 °C and re-suspended in 500 ul blocking buffer containing 5% goat serum (gibco, 16210072). CFTR antibodies were used at a dilution of 1:100 (2784 CF3 Abcam, CFTR-594 and CFTR-570 from CFTR Antibody Distribution Program). Staining was done for 30 minutes at RT in the dark. Cells were washed twice with permeabilization buffer and re-supended in 100 uL Alexa Fluor488 Goat anti-mouse antibody (Molecular Probes, A31619) for 20 minutes at RT. Finally, cells were washed twice with permeabilization buffer and once with FACS buffer before FACS assay.

### Apoptosis assay

MDMs were plated at a density of 1 × 10^6^/ml in 12 well plates. Apoptosis was measured by flow cytometry and fluorescence-activated cell sorting (FACS) analysis using APC Annexin V (Biolegend, 640920), prodidium iodide (BioLegend) and DAPI (Molecular Probes, D1306). MDMs were detached by Accutase solution, collected, washed, and re-suspended in 100 μl of Annexin V Binding Buffer (Biolegend 422201), and then stained with 5 μl of Annexin V and 0.4 µg/ml DAPI for 15 min at room temperature in the dark. As a positive control, untreated CF and non-CF MDMs were exposed to Thapsgargin (0.25 μM) for 12 hours. The percentages of viable and apoptotic cells was assessed using flow cytometry (BD LSR 11 Flow Cytometer; BD Bioscience).

### Polarization assay

MDMs were cultured in 12-well plates and exposed to an M1 polarization stimulus using *E*. *coli* LPS (Sigma, 20 ng/ml) plus recombinant human (rh)IFN-γ (Fisher, 20 ng/ml) and into M2 using rhIL-4 alone (20 ng/ml) for 48 hrs. Control cells without any stimulation were used for comparison. For phenotypic characterization of M1/M2 macrophages, cells were harvested by Accutase cell detach solution (Sigma), washed with FACS buffer and incubated in human Trustain FcX (Biolegend) at room temperature for 5 mins. Aliquots were first stained using PE anti-human CD_80_ & PE/Cy7 anti-human CD_11b_ or APC anti-human CD_126_ & APC/Cy7 anti-human CD_206_. Next, FITC anti-human CD_68_ intracellular staining was performed post fixation and permeabilization according to the manufacture’s recommendation. Stained cells were gated via viable CD_11b+_ subsets and identified by CD_68_CD_80_ (M1) and CD_163_CD_206_ (M2) markers using FlowJo (Tree Star).

### Phagocytosis assay

For phagocytosis of beads, Sphero^TM^ Ultra Rainbow Fluorescent beads (Spherotech Inc.) were opsonized with human serum at 37 °C for 45 min and layered onto MDMs in a ratio of 50:1 (beads to cells) for 18 hr, washed twice with PBS, and then imaged with an inverted fluorescence microscope or detached by Accutase for FACS analysis. For phagocytosis of bacteria, RFP-expressing *B*. *cenocepacia* were fixed with 4% paraformaldehyde for 30 min at room temperature, washed with PBS 5 times, and re-suspended in PBS containing 10% AB serum for 60 min at 37 °C. The serum-opsonized *B*. *cenocepacia* was incubated with MDMs at 50:1 (bacteria to cells) for 40 min at 37 °C, and detached for FACS analysis after PBS washing.

### Cytokine analysis

Human serum and MDM supernatants were treated with CFTR modulators or infected with *B*. *cenocepacia* for 18 hr, harvested, and stored at −20 °C until assayed. Cytokine levels were measured using the CBA human inflammatory cytokine kit assay (BD Biosciences Inc.) according to the manufacturer’s instructions. Assay limit of detection was 2.9 pg/ml.

### Statistical analysis

Statistical analyses were performed using GraphPad Prism software (version 6.1). Two sample unpaired t-tests were used for independent sample comparisons of CFTR expression. One-way ANOVA was used for apoptosis, polarization, phagocytosis, CFU, and cytokine analysis with post-hoc Tukey tests. Statistical significance was defined as a p value < 0.05. Age and gender matched healthy controls were used for comparison.

## Electronic supplementary material

Online Data Supplement

## Data Availability

The datasets generated during and/or analysed during the current study are available from the corresponding author on reasonable request.
