# Supplementary material for: Cystic fibrosis transmembrane conductance regulator (CFTR) modulators have differential effects on cystic fibrosis macrophage function
Source: Sci Rep. 2018 Nov 20;8:17066. doi: 10.1038/s41598-018-35151-7 (PMC6244248; doi:10.1038/s41598-018-35151-7)
Supplement: Supplementary file 1 — Online Data Supplement [file 41598_2018_35151_MOESM1_ESM.docx]

Cystic fibrosis transmembrane conductance regulator (CFTR) modulators have differential effects on cystic fibrosis macrophage function

**Shuzhong Zhang^1^, Chandra L. Shrestha^1^, and Benjamin T. Kopp^1,2^***

**ONLINE DATA SUPPLEMENT**

**Supplemental Table 1: CF patient Genotypes**

**Patient ID Genotype**

*CF no treatment group (n=24):*

CF09 F508del/F508del

CF17 F508del/F508del

CF19 F508del/ 2184insA

CF21 F508del/F508del

CF23 F508del/R1162X

CF29pre F508del/F508del

CF35 F508del/F508del

CF39 F508del/N1303K

CF47pre F508del/2789+5G>A

CF49pre 711+3A->G/F508del

CF51pre I507del/S945L

CF53pre G542X/S945L

CF61pre F508del/F508del

CF63pre F508del/F508del

CF65 F508del/F508del

CF67pre F508del/F508del

CF69pre F508del/F508del

CF81 F508del/F508del

CF93 F508del/F508del

CF95 DeltaF508/621+1G->T

CF101 F508del/F508del

CF107 F508del/F508del

CF109 F508del/R117H (5T/9T)

CF111 F508del/F508del

*Ivacaftor treatment group (n=10):*

CF07 G551D/F508del

CF11 R117H/F508del

CF13 G551D/F508del

CF37 R117H-7T/2789+5G>A

CF47post F508del/2789+5G>A

CF49post 711+3A->G/F508del

CF51post I507del/S945L

CF53post G542X/S945L

CF55post F508del/2789+5G>A
CF71post G542X/3849+10kbC->T

**
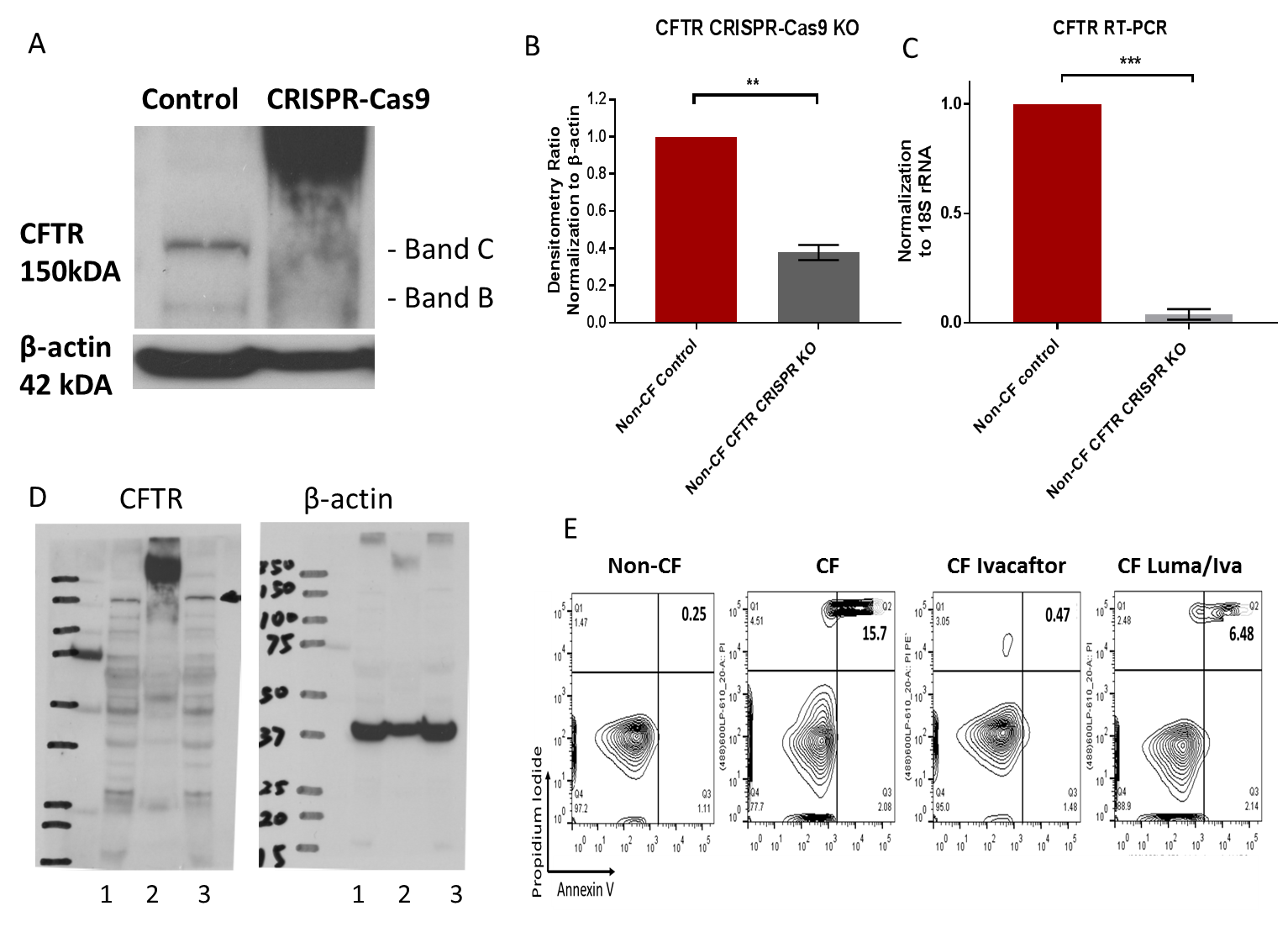
**

**Figure S1: CF3 antibody confirmation and apoptosis flow cytometry output.** A) Western blot for CFTR using CF3 antibody in non-CF human MDMs transfected with a CFTR CRISPR-Cas9 knockout plasmid or vehicle control. The loading control β-actin was run on the same gel. Full uncropped images are shown in Figure S1C. B) Summed densitometry for S1A normalized to non-CF CFTR expression, n=4, unpaired t-test. C) CFTR expression via RT-PCR in non-CF human MDMs transfected with a CFTR CRISPR-Cas9 knockout plasmid or vehicle control from S1A. D) Uncropped images for S1A. “1” = control, “2” = CRISPR-Cas9 KO, “3” = control not shown in S1A. E) Representative flow cytometry gating output for Figure 1D.

**
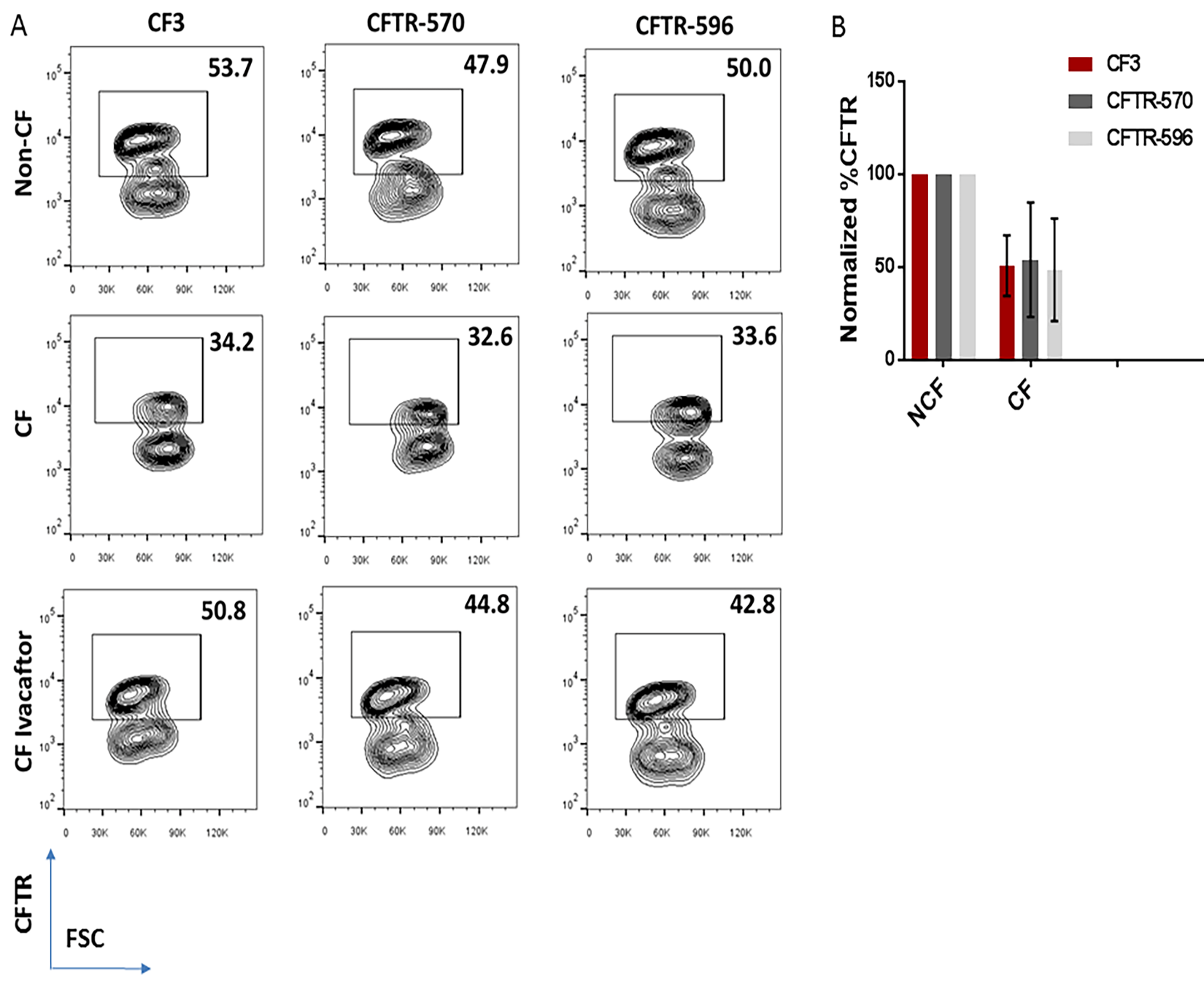
**

**Figure S2**: **CFTR antibody comparison via flow cytometry.** CF and non-CF macrophages were analyzed via flow cytometry for CFTR using 3 separate antibodies (CF3, CFTR-570, and CFTR-596). A) Representative gating strategy is shown for CF macrophages from a patient not on CFTR modulators in comparison to a non-CF patient and a CF patient on Ivacaftor with improved CFTR expression. B) Summed normalized %CFTR detected via flow cytometry assay for all 3 antibodies for non-CF MDMs and CF MDMs, n=3-6.

**
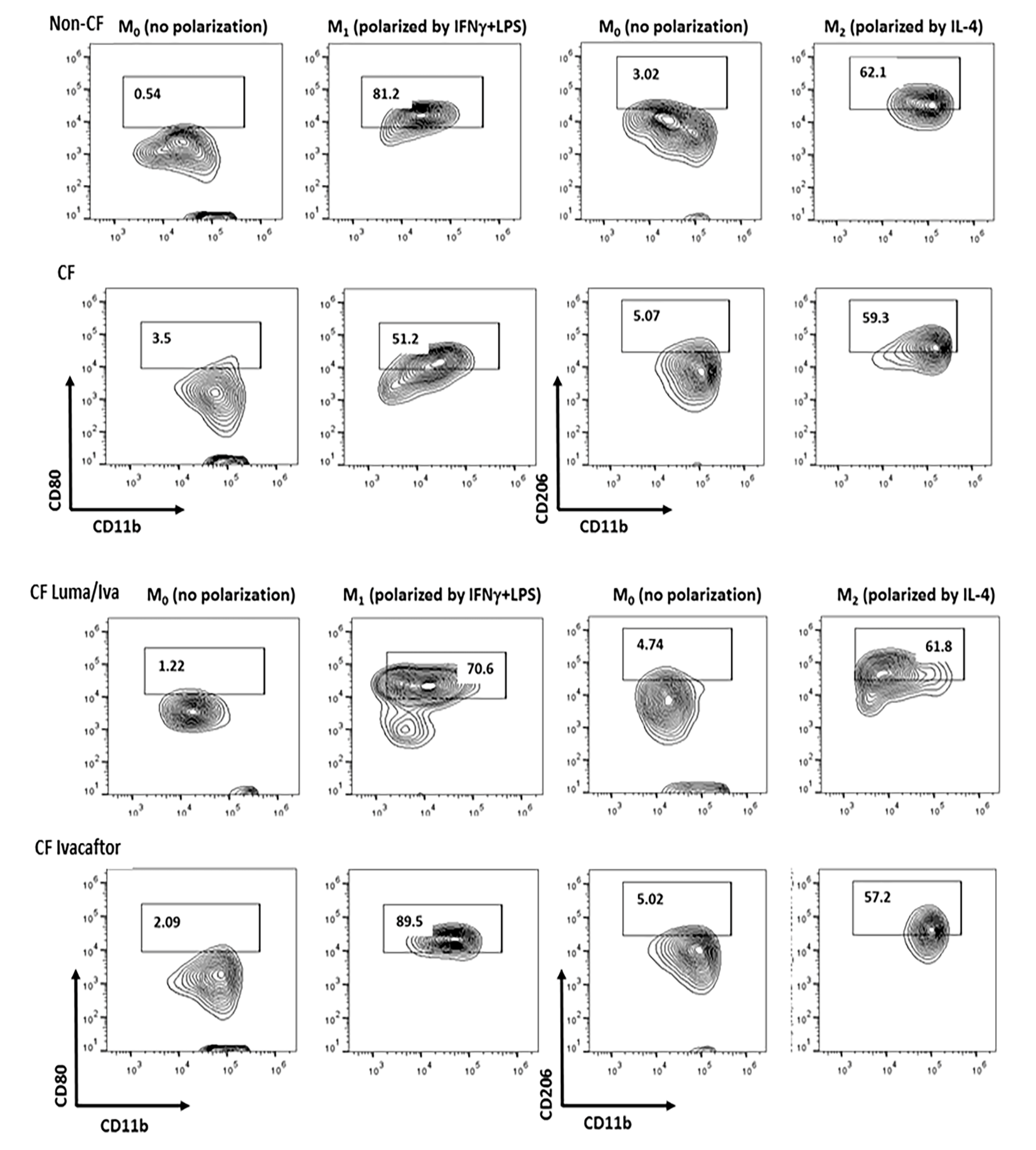
 Figure S3: Flow cytometry strategy for macrophage polarization.** CF and non-CF macrophages were stained with a panel of markers: CD_11b_CD_80_CD_68_ for M1 and CD_11b_CD_163_CD_206_ for M2. Cells were then gated via viable CD_11b+_ subsets, and identified by CD_80_CD_68_ (M1) and CD_163_CD_206_ (M2) markers using Flowjo (Tree Star).

**
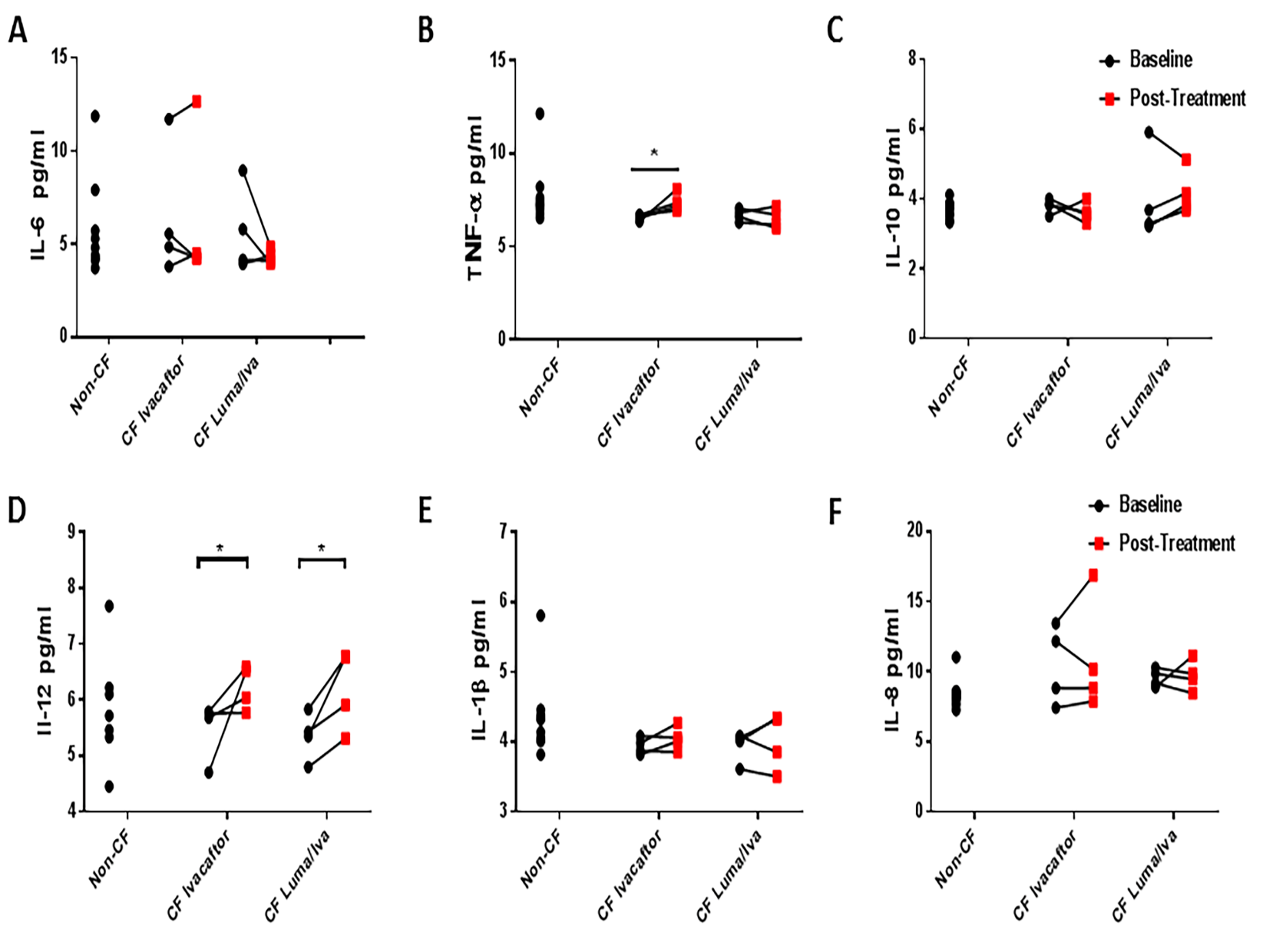
 Figure S4: Paired individual cytokine responses to CFTR modulation**. Multiplex cytokine assay analysis of serum from 8 CF and 10 non-CF patients for A) IL-6, B) TNF-α, C) IL-10, D) IL-12, E) IL-1β, and F) IL-8. Paired responses are shown for CF patients pre- and 3 months post-CFTR modulators. “*” denotes a p value < 0.05, one-way ANOVA with post-hoc Tukey.
